# Downregulation of CD132 in Colonic Adenomas and Cancer Is Associated with γδ T-Cell Loss, Increased Apoptosis, and Microsporidia Infection

**DOI:** 10.3390/cancers18142273

**Published:** 2026-07-15

**Authors:** Juan Carlos Andreu-Ballester, Cirilo Amorós-García, Salvador Benlloch-Pérez, Lorena Galindo-Regal, Carlos García-Ballesteros, Natalia Uribe, Ana Jiménez, Francisca López-Chuliá, Fernando Izquierdo, Elizabeth Valdivieso, Lucianna Vaccaro, Carmen del Aguila, Carmen Cuéllar, Carolina Hurtado-Marcos

**Affiliations:** 1 Foundation for the Promotion of Health and Biomedical Research in the Valencian Region (FISABIO—Public Health), 46020 Valencia, Spain; jcandreuballester@outlook.com; 2 Digestive Department, Arnau de Vilanova Hospital, 46015 Valencia, Spain; camorosg@gmail.com (C.A.-G.); salvador.benllochperez@uchceu.es (S.B.-P.); 3 Universidad Cardenal Herrera CEU, CEU Universities, 46115 Valencia, Spain; 4 Laboratory of Molecular Biology and Research Department, Arnau de Vilanova University Hospital, 46015 Valencia, Spain; loregal1610@gmail.com (L.G.-R.); garcia_carbar@gva.es (C.G.-B.); 5 Department of General and Digestive Surgery, Arnau de Vilanova University Hospital, 46015 Valencia, Spain; naturi62@gmail.com; 6 Pathology Department, Arnau de Vilanova University Hospital, 46015 Valencia, Spain; jimenez_ana@gva.es; 7 Hemathology Department, Arnau de Vilanova University Hospital, 46015 Valencia, Spain; lopez_frachu@gva.es; 8 Universidad CEU San Pablo, CEU Universities, Urbanización Montepríncipe, 28668 Boadilla del Monte, Spain; ferizqui@ceu.es (F.I.); elizabeth.valdiviesoblanco@ceu.es (E.V.); lucianna.vaccaro@ceu.es (L.V.); cagupue@ceu.es (C.d.A.); 9 Departamento de Microbiología y Parasitología, Universidad Complutense de Madrid, 28040 Madrid, Spain

**Keywords:** colorectal cancer, colon adenoma, CD132, γδ T cells, apoptosis, microsporidia, IL-7, immune dysregulation

## Abstract

**Background/Objectives:** Colorectal cancer commonly develops through an adenoma–carcinoma sequence, but the immune alterations accompanying this transition remain incompletely understood. Because we previously observed reduced γδ T cells, increased apoptosis, and frequent microsporidia infection in colorectal cancer, we investigated whether similar changes are already present in colonic adenomas. **Methods:** We studied 55 subjects, including 30 patients with colonic adenomas, 10 with colorectal cancer, and 15 healthy controls. Peripheral blood T-cell subsets and apoptosis were assessed by flow cytometry, tissue expression of IL-7, CD127, and CD132 was analyzed by RT-PCR, and microsporidia were detected in colonic tissues by immunofluorescence and real-time PCR. **Results:** γδ T cells were progressively reduced in colonic adenomas and further decreased in colorectal cancer, while apoptosis of both αβ and γδ T cells increased compared with healthy subjects. Tissue expression of CD132 was significantly downregulated in adenomas and colorectal cancer, whereas IL-7 expression was increased, particularly in adenomatous tissue, consistent with a compensatory mechanism attempting to preserve T-cell homeostasis despite reduced CD132 expression. Microsporidia prevalence rose from healthy controls to patients with colonic adenomas and colorectal cancer, and CD132 expression was more markedly reduced in microsporidia-positive colonic adenoma and cancer tissues. **Conclusions:** These findings indicate that altered IL-2 receptor-related signaling, γδ T-cell depletion, and increased apoptosis are already present at the adenoma stage and are associated with microsporidial infection. This pattern suggests a potential link between parasitic colonization and local immune dysfunction during colorectal tumorigenesis.

## Simple Summary

Colorectal carcinogenesis is a multistep process that begins in adenomatous lesions and is accompanied by local immune dysregulation. In this study, we analyzed whether altered immune signaling observed in Crohn’s disease and colorectal cancer is already present in colonic adenomas and whether it is associated with microsporidial infection. We found that CD132 expression was reduced in colonic adenomas and colorectal cancer, whereas γδ T cells progressively decreased and apoptosis increased from healthy tissue to colonic adenomas and cancer. Microsporidia showed a rising prevalence across this sequence and was associated with a more pronounced reduction in CD132.

## 1. Introduction

Since the 1970s, when areas of adenomatous tissue were identified in colorectal cancer and foci of cancer were found in larger colonic adenomas, it has been suggested that colorectal cancer could evolve from adenomatous polyps [1]. In addition, natural history studies of patients with polyps who refused excision demonstrated that one third of them developed colorectal cancer within 20 years [2,3]. Together with the clear relationship between colorectal cancer and familial adenomatous polyposis through germline mutations in the adenomatous polyposis coli (APC) tumor suppressor gene, these findings demonstrate that adenomatous polyps are the precursors of the vast majority of colorectal cancers [4,5,6]. Somatic mutations in intestinal cells are common in healthy individuals and are far more numerous in patients with colonic adenomas and cancer. Therefore, attempts have been made to classify colorectal cancer according to gene expression profiles into molecular subtypes (CMS1–4), each associated with distinct molecular characteristics and clinical outcomes [7,8,9].

In addition to genetic alterations, increasing evidence indicates that progressive remodeling of the colorectal tumor microenvironment, including defects in immune surveillance and immune homeostasis, plays a critical role during tumor initiation and progression [10].

Beyond genetic alterations, interactions between the intestinal microbiota and the immune system are increasingly recognized as important contributors to colorectal carcinogenesis [11].

Microsporidia are worldwide parasites that mainly affect immunocompromised individuals, especially those with advanced HIV or other severe immune suppression. They commonly cause gastrointestinal disease with chronic diarrhea and can be life-threatening in severe cases [12,13].

Recently, we have demonstrated that more than 40% of patients with colorectal cancer harbor microsporidia in their tumor tissues, indicating a significantly higher prevalence of microsporidiosis than in healthy control subjects. In addition, serum levels of anti-microsporidia antibodies in patients with colorectal cancer were significantly higher than those in control subjects [14].

In addition, we previously demonstrated a significant decrease in γδ T cells and an increase in apoptosis in patients with colorectal cancer [15]. These findings are consistent with recent evidence highlighting the critical role of γδ T cells in colorectal cancer immunity and tumor progression [16]. This deficiency was also demonstrated by us in patients with Crohn’s disease (CD) [17]. This disease has been associated with a higher incidence of colorectal cancer, extraintestinal cancer and microsporidia infection [18,19,20]. In searching for a possible cause of this decrease in γδ T cells in CD, we discovered lower gene expression of the IL-2 receptor γ subunit (CD132) in tissues from patients with CD, which was associated with reduced γδ T-cell levels and increased apoptosis [21]. The similarity of the immunological findings in both conditions, suggesting a shared mechanism of action, has led us to propose the present research study with the aim of investigating whether the altered signaling observed in CD and colorectal cancer is already present in patients with colon polyps—possible precursors of malignant transformation—and to analyze its prevalence and relationship with microsporidia.

## 2. Materials and Methods

### 2.1. Subjects of Study, Clinical Samples and Ethics Statement

Fifty-five patients were recruited for this study: 30 with colonic adenomas (AD), 10 with colorectal cancer (CC), and 15 healthy controls (HCs). All participants were evaluated in the colonoscopy unit of the Department of Gastroenterology at Arnau de Vilanova Hospital in Valencia (Spain) over a six-month period in 2025 (January to June). Blood samples were collected for the analysis of T-cell subsets, and tissue samples were obtained for the study of gene expression of interleukin-7, CD127, and CD132, as well as for the detection of microsporidia.

Patients and healthy controls were excluded from the study if they met the following criteria: known immunodeficiency; infectious, inflammatory or autoimmune disease; and specific active antitumor or immunosuppressive treatment, as well as the application of any vaccine during at least the last six months.

The Hospital Research Ethics Committee approved the study, and each patient signed an informed consent document. This epidemiological investigation was conducted in accordance with fundamental ethical principles, in particular those contained in the Charter of Fundamental Rights of the European Union and the European Convention on Human Rights and its Additional Protocols. All participants attested to their participation in this clinical study with written informed consent. Consent was evaluated and approved by the Research Ethics Committee of the Arnau de Vilanova Hospital (CEIm), in accordance with the recommendations of the Spanish Bioethics Committee and the Spanish legislation on biomedical research (Law No. 14/2007 of 3 July) and protection of personal data (Spanish Law No. 3/2018 and European Law UE676/2018). These laws provide that access to clinical records for judicial, epidemiological, public health, research, or educational purposes requires separation of identifiable patient data from clinical and health data. Anonymity is thus guaranteed.

### 2.2. Immunofluorescence Antibody Test (IFAT)

#### 2.2.1. Deparaffination and Imprints

Tissue sections were deparaffinized by sequential immersion for 10 min in xylene, xylene–ethanol, absolute ethanol, 90% ethanol, 70% ethanol, and distilled water, as previously described [20]. Deparaffinized sections and imprints were air-dried and fixed with methanol–acetone. Sample areas were delimited using a DAKOPEN^®^ marker (Biolabs) (Agilent Technologies, Santa Clara, CA, USA) to confine IFAT reagents.

#### 2.2.2. Assay

Two primary monoclonal antibodies (mAbs) were employed. mAb 2C2 (murine IgG2a) recognizes the exospore wall and developmental stages of *Encephalitozoon* spp. [22], whereas mAb 6E52D9 (murine IgG2a) specifically recognizes the exospore wall of *Enterocytozoon bieneusi* [23].

Undiluted mAb supernatants were applied to tissue sections or imprints and incubated at 37 °C for 60 min in a humid chamber. Slides were washed three times with distilled water and air-dried at room temperature.

A fluorescein isothiocyanate (FITC)-conjugated secondary antibody (Sigma, St. Louis, MO, USA), diluted according to the manufacturer’s instructions, was then applied and incubated at 37 °C for 60 min. Slides were subsequently washed three times with distilled water and air-dried in the dark at room temperature.

Slides were mounted with PVA/Dabco, coverslipped, and examined at 40× magnification using a Nikon immunofluorescence microscope (Nikon Corporation, Tokyo, Japan). Positive controls consisted of homologous microsporidia species corresponding to each monoclonal antibody. Slides were stored at 4 °C in the dark until analysis.

Because IFAT detects morphologically intact microsporidial spores and developmental stages directly within tissue sections, whereas PCR detects parasite DNA extracted from tissue, both techniques were used as complementary diagnostic approaches rather than alternative methods. This combined strategy increases the likelihood of detecting tissue infection and allows for the identification of biologically distinct situations that may not be detected by a single technique [14].

### 2.3. DNA Extraction and Detection of Microsporidia by Multiplex Real-Time PCR

Genomic DNA was extracted from colon biopsy specimens obtained from patients using the DNeasy Blood and Tissue Kit (Qiagen, Hilden, Germany), according to the manufacturer’s instructions. Detection of microsporidia was performed by real-time PCR using SYBR Green chemistry, following the protocol described by Polley et al. [24] with modifications by Andreu-Ballester et al. [19]. Amplification was carried out using the primers MsRTF (5′-CAGGTTGATTCTGCCTGACG-3′) and MsRTR (5′-CCATCTCTCAGGCTCCCTCT-3′), which target the SSU rRNA gene of *Encephalitozoon intestinalis*, *Encephalitozoon cuniculi*, *Encephalitozoon hellem*, and *Enterocytozoon bieneusi*. Each 20 µL reaction contained 10 pmol of each primer and 5 µL of template DNA. Thermal cycling conditions consisted of an initial denaturation at 95 °C for 10 s, followed by 35 cycles of denaturation at 95 °C for 10 s, annealing at 60 °C for 20 s, and extension at 72 °C for 20 s. All samples were analyzed in duplicate.

PCR detects microsporidial DNA independently of the presence of morphologically identifiable spores and may therefore identify infected tissues in which parasite DNA is present despite the absence of detectable spores in the histological section analyzed by IFAT. Conversely, IFAT may detect morphologically preserved spores in samples with degraded or insufficient DNA for amplification. Therefore, results obtained by both techniques were interpreted as complementary rather than requiring complete concordance [14].

### 2.4. Gene Expression of IL-7, IL-7 Receptor α (CD127) and IL-2 Receptor Subunit γ (CD132) in Tissues

The samples were homogenized with 1 mL Trizol^®^ Reagent (Ambion, Life Technologies, Austin, TX, USA) for RNA isolation following the manufacturer’s instructions. RNA concentration was measured using a GE NanoVue Spectrophotometer (GE Healthcare Life Sciences, Coventry, England). A reverse transcription reaction was performed with 1 μg extracted RNA using the Thermo Scientific RevertAid H Minus First Strand cDNA Synthesis kit (Thermo Fisher Scientific, Waltham, MA, USA). The resulting cDNA was amplified and subjected to quantitative PCR (qPCR) in a Gene Amp 5700 (PE Biosystems, Framingham, MA, USA). The primer sequences are shown in Table A1. The relative expression of each gene was normalized to the housekeeping gene β-actin and calculated using the 2^-ΔΔCt method.

### 2.5. Analysis of γδ and αβ T Cells and Apoptosis Evaluation

Phenotypic and functional analyses of γδ and αβ T cells were performed in peripheral blood and intestinal tissue samples using multiparameter flow cytometry. Cells were stained with the following monoclonal antibodies: anti-TCR pan αβ-PE, anti-TCR pan γδ-PE and -FITC, CD3-PC5 and -ECD, CD4-PC7, CD8-PC7 and -FITC, CD5-FITC, CD19-PC7, CD56-PC7 and -PE, and CD45-ECD (Beckman Coulter, Brea, CA, USA). Data acquisition was performed on a Navios flow cytometer (Beckman Coulter), with a total of 100,000 events collected per sample. Data were subsequently analyzed using Kaluza software version 2.1 (Beckman Coulter, Brea, CA, USA).

**Figure A1:**
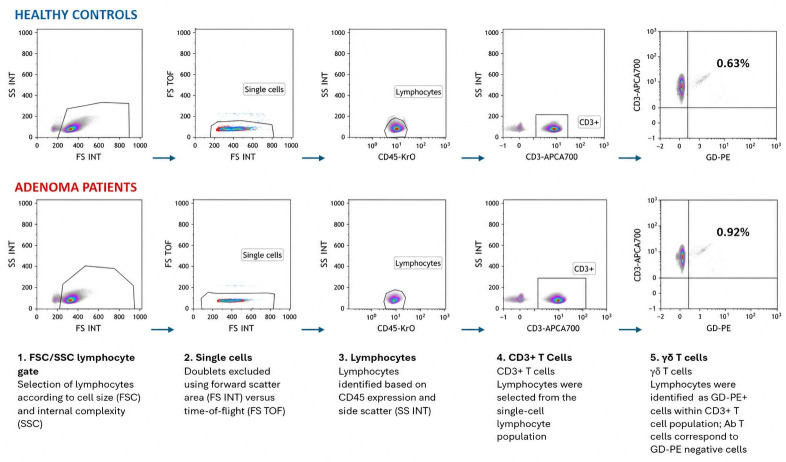
Gating strategy used to identify T-cell subsets in healthy controls and patients with colon disease.

Apoptosis in peripheral blood lymphocytes was assessed using the Annexin V-FITC/7-AAD apoptosis detection kit (Beckman Coulter) according to the manufacturer’s instructions. This assay is based on the binding of Annexin V to phosphatidylserine exposed on the outer leaflet of the plasma membrane during early apoptosis, and the selective incorporation of 7-amino-actinomycin D (7-AAD) into DNA at guanine–cytosine base pairs in late apoptotic or necrotic cells. The gating strategy used to identify T-cell subsets in healthy controls and patients with colon disease is shown in Figure A1.

### 2.6. Statistical Analysis

The ANOVA test with Bonferroni correction was used for the comparison of means in the three groups. The Mann–Whitney U test was applied to compare the mean values between two quantitative variables. Correlations between interleukin-7, CD132, CD127 gene expression and T-cell subsets were assessed using Spearman’s correlation coefficient. Statistical analyses were conducted with the Statistical Package for the Social Sciences (SPSS version 19.0; SPSS Inc., Chicago, IL, USA), and graphical representations were generated using GraphPad Prism version 8.0.0 (GraphPad Software, San Diego, CA, USA).

## 3. Results

### 3.1. Progressive Immune Dysregulation in Colonic Adenoma and Colorectal Cancer is Associated with γδ T-Cell Loss and Increased Apoptosis

To investigate whether the immune alterations previously described in colorectal cancer are already present at the adenomatous stage, we first analyzed circulating αβ and γδ T-cell populations in healthy controls (HCs), patients with colonic adenomas (AD), and patients with colorectal cancer (CC).

**Figure 1:**
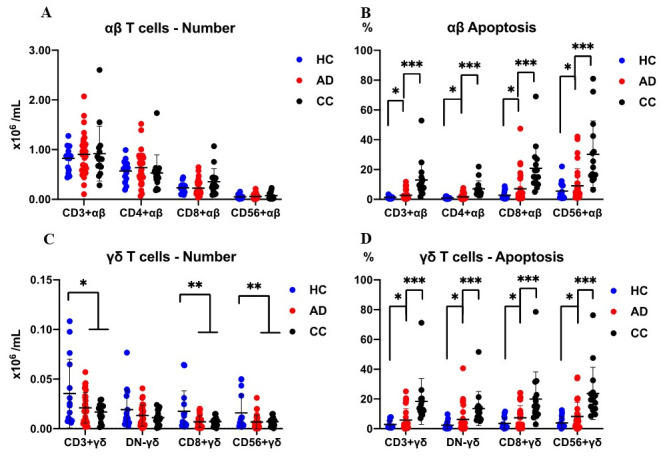
Progressive alterations in circulating T-cell subsets during colorectal disease progression. (**A**) αβ T-cell counts. (**B**) Apoptosis of αβ T cells. (**C**) γδ T-cell counts. (**D**) Apoptosis of γδ T cells in healthy controls (HC), Colonic adenoma patients (AD), and colorectal cancer patients (CC). Values are expressed as mean ± SD. ANOVA with Bonferroni correction was used for multiple comparisons. * *p* < 0.05; ** *p* < 0.01; *** *p* < 0.001.

No significant differences were observed in αβ T-cell counts among the three groups (Figure 1A). In contrast, γδ T cells showed a progressive reduction from healthy controls to colonic adenoma patients and were further decreased in colorectal cancer patients (Figure 1C), suggesting a gradual impairment of this lymphocyte subset during colorectal disease progression.

In parallel, apoptosis rates increased progressively along the adenoma–carcinoma sequence. Both αβ and γδ T cells from AD and CC patients displayed significantly higher apoptosis compared with HCs (Figure 1B,D). Notably, the increase was more pronounced in γδ T cells, supporting the existence of a progressive immune imbalance associated with colorectal tumorigenesis.

### 3.2. Altered IL-7/CD127/CD132 Signaling Is Already Present in Adenomatous Lesions

Given the progressive reduction in γδ T cells, we next evaluated the expression of genes involved in IL-7 signaling, a pathway essential for T-cell survival and homeostasis.

**Figure 2:**
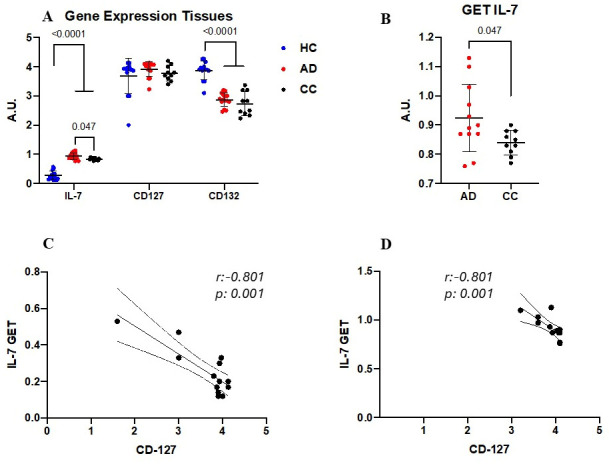
Alterations in IL-7 signaling-related gene expression in colorectal disease. Tissue expression of IL-7, CD127 and CD132 in healthy controls (HC), colonic adenoma patients (AD) and colorectal cancer patients (CC) (**A**). Comparison of IL-7 expression between AD and CC tissues (**B**). Correlation between IL-7 and CD127 expression in healthy controls and colonic adenoma patients (**C**,**D**). Values are expressed in arbitrary units (A.U.). Mann–Whitney U test was used. Correlation between IL-7 and CD127 gene expression in (**C**) healthy controls and (**D**) colonic adenoma patients. Spearman’s r test was used. ANOVA with Bonferroni correction was used for the comparison of means in the three groups. Dashed lines indicate the 95% confidence intervals of the regression line.

IL-7 gene expression in tissues was significantly increased in colonic adenoma samples compared with healthy subjects, remaining elevated in colorectal cancer tissues although at slightly lower levels than in colonic adenomas (Figure 2A). In contrast, expression of the IL-2 receptor common γ-chain (CD132), a key component of the IL-7 receptor complex, was significantly reduced both in colonic adenoma and colorectal cancer tissues (Figure 2B).

CD127 expression showed a less pronounced variation among groups; however, significant correlations between IL-7 and CD127 gene expression were observed in healthy controls and colonic adenoma patients (Figure 2C,D), suggesting preservation of partial IL-7 responsiveness at early stages of disease.

Overall, these findings indicate that dysregulation of the IL-7/CD132 axis appears early during colonic adenoma development and persists during malignant progression.

### 3.3. Microsporidia Prevalence Increases Along the Adenoma–Carcinoma Sequence

We next investigated the prevalence of microsporidia in colorectal tissues from HC, AD, and CC patients.

**Figure 3:**
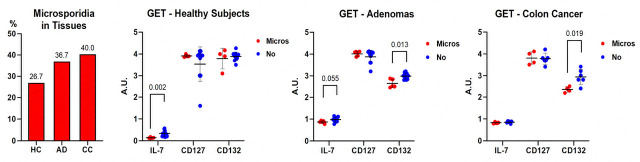
Prevalence of microsporidia (**A**) and gene expression in tissues (GET) of IL-7, CD127 and CD132 in healthy controls (HCs) (**B**), colonic adenoma (AD) (**C**) and colorectal cancer (CC) (**D**).

Microsporidia were detected in 26.7% of healthy subjects, 36.7% of colonic adenoma samples, and 40.0% of colorectal cancer tissues (Figure 3A). Although these differences did not reach statistical significance, a progressive increase in microsporidia positivity was observed from healthy mucosa to adenomatous lesions and malignant tissues.

This gradual enrichment of microsporidia across the adenoma–carcinoma sequence supports a potential association between microsporidial colonization and colorectal disease progression.

### 3.4. Microsporidia-Positive Tissues Show Enhanced CD132 Downregulation

To determine whether microsporidia infection was associated with alterations in immune signaling, we compared IL-7 pathway-related gene expression according to microsporidia positivity.

In healthy subjects, IL-7 expression tended to be lower in tissues harboring microsporidia (Figure 3B). More importantly, colonic adenoma and colorectal cancer tissues positive for microsporidia exhibited a marked reduction in CD132 expression compared with microsporidia-negative samples (Figure 3C,D). This association was not observed in healthy subjects.

**Figure A2:**
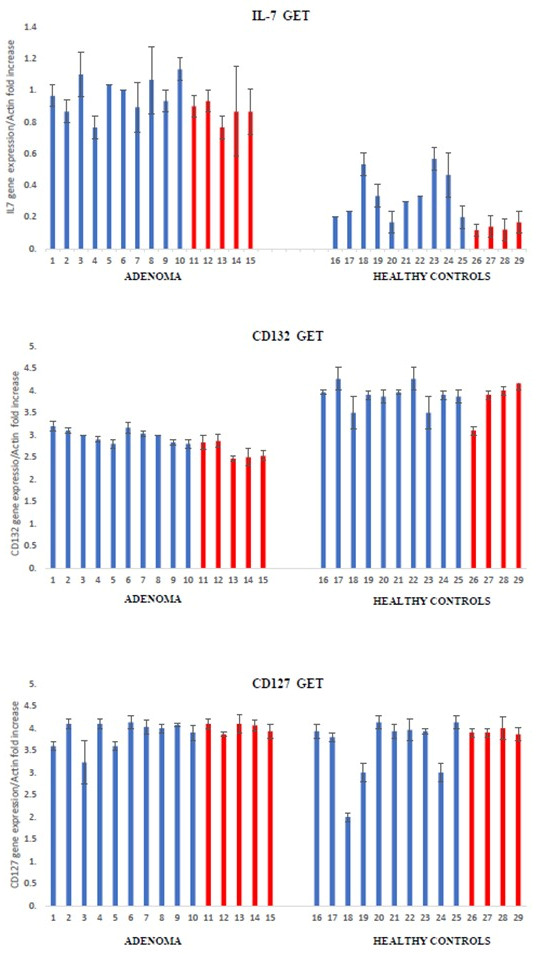
Gene expression of IL-7, CD132, and CD127 determined by RT-PCR. The relative expression of each gene was normalized to the housekeeping gene β-actin. Cases positive for microsporidia are shown in red.

**Figure A3:**
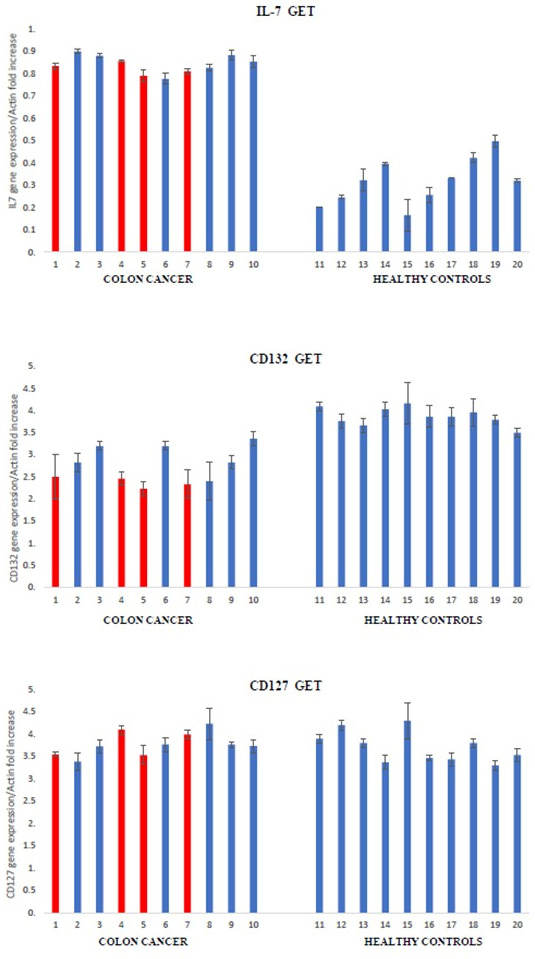
Gene expression of IL-7, CD132, and CD127 determined by RT-PCR. The relative expression of each gene was normalized to the housekeeping gene β-actin. Cases positive for microsporidia are shown in red.

These findings suggest that microsporidia presence is specifically associated with impaired CD132-mediated immune signaling in premalignant and malignant colorectal tissues (Figure A2 and Figure A3).

### 3.5. Peripheral T-Cell Apoptosis Is Associated with Tissue Microsporidia Positivity

Finally, we analyzed whether the presence of microsporidia in tissues was associated with systemic alterations in circulating T-cell populations.

**Figure 4:**
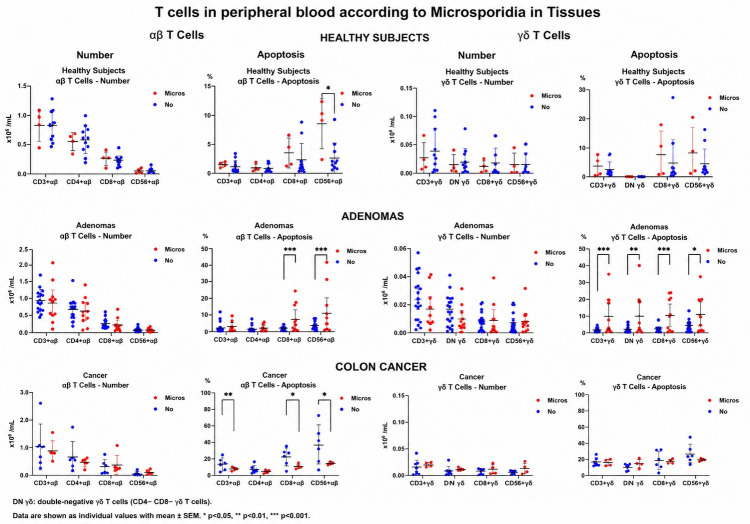
Number (**columns 1 and 3**) and apoptosis (**columns 2 and 4**) of T cells (αβ and γδ) in peripheral blood according to microsporidia in tissues from healthy controls (HCs, *n* = 15) and adenoma (AD, n = 30) and colorectal cancer (CC, n = 10) patients. * *p* < 0.05, ** *p* < 0.01 and *** *p* < 0.001.

No significant differences were observed in αβ or γδ T-cell counts according to tissue microsporidia positivity (Figure 4, columns 1 and 3). However, apoptosis analysis revealed distinct patterns depending on disease stage.

In colonic adenoma patients, microsporidia-positive tissues were associated with increased apoptosis of circulating αβ T cells. Conversely, in colorectal cancer patients, lower apoptosis levels were observed in peripheral T cells from subjects harboring microsporidia in their tissues (Figure 4, columns 2 and 4).

Together, these results suggest that microsporidia may influence systemic immune regulation differently during premalignant and malignant stages of colorectal disease.

## 4. Discussion

This study is the first to investigate the gene expression of the common γ-chain of the interleukin-2 receptor (CD132) in colorectal cancer tissues and its relationship with microsporidiosis. In a previous study, we observed a significant reduction in γδ T cells—particularly CD8^+^ γδ T cells—in patients with colorectal cancer, along with a marked increase in apoptosis in both αβ and γδ T cells compared with healthy controls [15]. More recently, we also demonstrated an increased prevalence of microsporidia in colorectal cancer tissues [14]. Together, these findings suggest a potential link between impaired T-cell homeostasis, altered CD132 expression, and susceptibility to microsporidial infection in colorectal cancer.

An important novel finding of this study is that CD132 downregulation is already detectable at the adenoma stage, indicating that disruption of common γ-chain cytokine signaling occurs before the development of invasive colorectal cancer. Because CD132 is shared by the receptors for IL-2, IL-4, IL-7, IL-9, IL-15, and IL-21, its reduced expression may impair multiple pathways required for lymphocyte survival, proliferation, and immune surveillance. This early alteration provides a plausible mechanistic explanation for the progressive depletion of γδ T cells and the increased apoptosis observed during colorectal tumorigenesis. Therefore, CD132 downregulation may represent not only a biomarker of early immune dysfunction but also a potential contributor to adenoma progression.

In addition, we also reported a reduction in γδ T cells in Crohn’s disease and its association with microsporidia [17,20]. More recently, we demonstrated a significant decrease in the gene expression of the common γ-chain of the interleukin-2 receptor (CD132) in tissues, suggesting a potential role in the reduction in this cellular subset [21].

The progression of colonic adenomatous polyps to colorectal cancer is well established [2,3]. Therefore, in the present study we analyzed the previously described immunological profile in order to identify similar alterations in adenomatous polyps. Our results show that the number of γδ T cells begins to decrease in patients with colonic adenomas and declines even further in patients with colorectal cancer. In contrast, apoptosis displays an opposite pattern, increasing in patients with colonic adenomas and becoming more pronounced in those with cancer.

The progressive loss of γδ T cells together with enhanced apoptosis suggests that impairment of local immune surveillance may occur early during colonic adenoma formation.

Although parallel alterations were observed in colonic tissue and peripheral blood, the present study was not designed to establish a mechanistic relationship between these compartments. Therefore, whether local immune dysregulation and microsporidia infection directly contribute to the systemic T-cell alterations observed remains to be determined. Future longitudinal and functional studies will be necessary to clarify this relationship.

Interestingly, gene expression of the interleukin-2 receptor common γ-chain (CD132) is reduced both in colonic adenomas and in colorectal cancer patients. Notably, IL-7 gene expression is increased in adenomatous tissues and, although it is also elevated in cancer, this increase appears to be less marked.

CD132, also known as the common γ-chain (γc), is a critical component shared by several cytokine receptors, including those for IL-2, IL-4, IL-7, IL-9, IL-15, and IL-21. This receptor subunit plays an essential role in T-cell survival, proliferation, differentiation, NK-cell biology, and overall lymphocyte homeostasis [25,26]. Alterations in γc-dependent signaling pathways have been associated with profound immune dysfunction and impaired cellular immunity [27,28].

It should be noted that CD132 is not specific to the IL-7 receptor but constitutes the common γ-chain shared by the receptors for IL-2, IL-4, IL-7, IL-9, IL-15, and IL-21. Consequently, the reduced CD132 expression observed in colonic adenomas and colorectal cancer may have broader biological implications than impaired IL-7 signaling alone, potentially affecting multiple cytokine pathways involved in lymphocyte survival, proliferation, differentiation, and immune surveillance. However, the present study evaluated receptor expression and was not designed to assess downstream signaling events such as JAK/STAT activation. Future functional studies will be required to determine the impact of CD132 downregulation on γ-chain-dependent cytokine signaling during colorectal tumorigenesis.

Therefore, in the present study, the reduced expression of CD132 observed in colonic adenomas and colorectal cancer tissues may reflect a broader defect in cytokine-dependent immune regulation within the colorectal microenvironment, potentially contributing to defective immune surveillance and progressive tumor development.

We speculate that the upregulation of IL-7 may represent a compensatory attempt to promote the generation of γδ T cells; however, this mechanism may be ineffective due to altered expression of the IL-7 receptor, a heterodimer composed of CD127 and CD132. The deficient gene expression of one of its key components, CD132, could impair the signaling required for the production and proliferation of γδ T cells.

The concordance observed between IL-7 gene and protein expression supports the biological relevance of the transcriptional findings. However, similar protein-level validation was not performed for CD127 or CD132. Therefore, the observed changes in receptor expression should be interpreted at the transcriptional level, and future studies should confirm whether these alterations are also reflected at the protein level.

Future studies should validate these transcriptional findings in prospectively recruited patient cohorts using freshly collected peripheral blood and colonic tissue samples specifically processed for cellular and protein analyses. Such studies, which will require dedicated sample collection protocols and new ethical approvals, will enable detailed characterization of CD127 and CD132 expression in γδ and αβ T-cell subsets by flow cytometry, as well as assessment of their protein expression and spatial distribution within the colonic mucosa by immunofluorescence or other complementary approaches. These investigations will be essential to clarify the cellular mechanisms underlying the associations observed in the present study and to further define the role of the IL-7/CD132 axis during colorectal tumorigenesis and microsporidia infection.

While tissue availability precluded the assessment of CD132 protein distribution by immunohistochemistry, previous studies have established that CD132 (the common cytokine receptor γ-chain) is constitutively expressed in the intestinal mucosa, predominantly by mucosal T lymphocytes, including intraepithelial and lamina propria T cells, as well as by intestinal epithelial cells. Given that our flow cytometric analyses demonstrated a concurrent reduction in γδ T cells, it is plausible that the decreased CD132 transcript levels observed in our study reflect, at least in part, the depletion of these mucosal T-cell populations. Nevertheless, a more generalized downregulation involving epithelial cells or other immune cell subsets cannot be excluded and should be addressed in future studies using immunohistochemistry or spatial transcriptomic/proteomic approaches.

Our findings should also be interpreted within the broader context of the immunosuppressive colorectal tumor microenvironment. Although the present study focuses on alterations in the IL-7/CD132 axis and γδ T-cell homeostasis, recent evidence indicates that additional immunosuppressive mechanisms contribute to colorectal tumorigenesis. In particular, WNT2 signaling has been shown to promote the expansion and immunosuppressive activity of myeloid-derived suppressor cells (MDSCs), thereby facilitating immune evasion and tumor progression. These observations support the concept that colorectal cancer develops through the interplay of multiple complementary mechanisms of immune dysregulation. In this context, the reduced CD132 expression and γδ T-cell depletion observed in our study may represent an additional component of this complex immunosuppressive network rather than an isolated mechanism [29].

However, the present study does not establish whether this alteration is directly responsible for the observed defects in γδ T-cell homeostasis or reflects a consequence of the evolving tumor microenvironment.

A limitation of the present study is the lack of functional analyses to determine whether reduced CD132 expression directly impairs γ-chain cytokine signaling. Future studies should evaluate IL-7- and IL-15-dependent STAT5 activation, cytokine responsiveness, and the consequences of experimentally restoring CD132 expression on γδ T-cell survival and function. Such experiments will be necessary to establish whether CD132 downregulation is a causal driver of immune dysfunction or a consequence of the altered immune microenvironment during colorectal tumorigenesis.

Finally, we must acknowledge some limitations in our study. While our sample size for the adenoma cohort (n = 30) was sufficient to detect significant early immunological shifts, the colorectal cancer cohort was limited to ten patients. This small sample size inherently restricts the statistical power of our study, particularly for sub-analyses regarding microsporidia status within the cancer group. Consequently, these specific findings should be interpreted with caution and regarded as exploratory or hypothesis-generating. Future larger-scale, prospective multicenter studies are required to validate the robustness of the association between microsporidial infection, CD132 downregulation, and local immune dysfunction in advanced colorectal malignancy.

It has been demonstrated that microsporidia are capable of directly increasing the cellular mutation rate of the host [30]. In addition, evidence indicates that microsporidia are able to proliferate inside host cells while avoiding activation of the p53-dependent apoptotic response [31].

The present study was not designed to compare the biological effects of individual microsporidia species. Although Encephalitozoon spp. and Enterocytozoon bieneusi differ in their biology and pathogenicity, both were considered together as indicators of tissue microsporidia infection. Future studies including larger numbers of infected samples will be required to determine whether species-specific differences influence immune alterations during colorectal tumorigenesis.

Regarding microsporidia, a clear trend toward increased prevalence is observed in patients with colonic adenomas, which becomes even more pronounced in those with colorectal cancer. Notably, expression of the interleukin-2 receptor common γ-chain (CD132) is significantly reduced in both colonic adenoma and colorectal cancer patients compared with healthy subjects, and this reduction is even more marked in individuals harboring microsporidia. Together, these findings suggest a strong association between microsporidial infection and impaired immune signaling, supporting the hypothesis that microsporidia may contribute to an immunologically permissive microenvironment associated with colorectal tumor progression.

Because this study has a cross-sectional design, the observed association between microsporidia infection and reduced CD132 expression should not be interpreted as evidence of a causal relationship. An alternative explanation is that the altered immune microenvironment associated with colonic adenoma formation and colorectal cancer facilitates colonization or persistence of microsporidia. Conversely, microsporidia may contribute to local immune dysregulation through mechanisms that remain to be elucidated. These possibilities are not mutually exclusive and should be addressed in future longitudinal and mechanistic studies.

## 5. Conclusions

In summary, immunological alterations similar to those observed in colorectal cancer are already present at the adenoma stage. To our knowledge, this study provides the first evidence that CD132 downregulation is already present at the adenoma stage and is associated with γδ T-cell depletion, increased apoptosis, and microsporidia infection. These findings identify an early pattern of immune dysregulation during the adenoma–carcinoma sequence and support the potential value of CD132 as a biomarker of early immune alterations in colorectal tumorigenesis. Future functional studies will be required to elucidate the mechanistic role of CD132 in this process, as we believe, and to evaluate its potential as a therapeutic target in colorectal cancer.

However, the present study identifies associations rather than causal mechanisms, and further longitudinal and functional studies will be required to establish the mechanistic relationships among CD132 downregulation, immune dysfunction, microsporidia infection, and colorectal tumorigenesis.

## Data Availability

The raw data supporting the conclusions of this article will be made available by the authors on request.

## Appendix A

**Table A1:** Primer sequences of IL-7, IL-7 receptor, and reference genes used for RT-PCR analysis.

Gene	Forward Primer (5′→3′)	Reverse Primer (5′→3′)
IL7	GAG TGA CTA TGG GCG GTG AGA G	GAT GCT ACT GGC AAC AGA ACA AGG
CD127 (IL7Rα)	CAC CCA AGT CAA TGC CTT TT	TGA GCA TTC AGT AGC CAT GC
CD132 (IL2Rγ)	TAT GTG CTC CTG CTC CCT CT	ACC CCC ACA CTC TGT CTG TC
ACTIN	CACCATTGGCAATGAGCGGTTC	AGGTCTTTGCGGATGTCCACGT

**Table A2:** Antibodies used for flow cytometry analysis.

Antibody	Clone	Beckman Coulter Catalog Number	RRID
TCR pan γδ-PE	IMMU510	B49176 (CE)/IM1418U (ASR)	Not available
TCR pan γδ-FITC	IMMU510	B49175 (CE)/IM1571U (ASR)	Not available
TCR pan γδ-PC5	IMMU510	IM2662	RRID:AB_131175
CD3-PC5	UCHT1	A07749 (CE)/IM2635U (ASR)	RRID:AB_2827419
CD4-PC7	13B8.2	6607101	Not available
CD8-PC7	B9.11	6607102	Not available
CD8-FITC	B9.11	A07756 (CE)/IM0451U (ASR)	Not available
CD5-FITC	BL1a	Not available	Not available
CD19-PC7	J3-119	IM3628 (CE)/IM3628U (ASR)	Not available
CD56-PC7	N901	A21692 (CE)/A51078 (ASR)	Not available
CD56-PE (RD1)	N901	6603067	Not available
CD45-ECD	J33	A07784 (CE)/710U (ASR)	Not available

## References

[1] Morson B. (1974). The polyp-cancer sequence in the large bowel. Proc. R. Soc. Med..

[2] Stryker S.J., Wolff B.G., Culp C.E., Libbe S.D., Ilstrup D.M., MacCarty R.L. (1987). Natural history of untreated colonic polyps. Gastroenterology.

[3] Huck M.B., Bohl J.L. (2016). Colonic polyps: Diagnosis and surveillance. Clin. Colon Rectal Surg..

[4] Kinzler K.W., Nilbert M.C., Vogelstein B., Bryan T.M., Levy D.B., Smith K.J., Preisinger A.C., Hamilton S.R., Hedge P., Markham A. (1991). Identification of a gene located at chromosome 5q21 that is mutated in colorectal cancers. Science.

[5] Miyaki M., Konishi M., Kikuchi-Yanoshita R., Enomoto M., Igari T., Tanaka K., Muraoka M., Takahashi H., Amada Y., Fukayama M. (1994). Characteristics of somatic mutation of the adenomatous polyposis coli gene in colorectal tumors. Cancer Res..

[6] Rohrbeck A., Borlak J. (2009). Cancer genomics identifies regulatory gene networks associated with the transition from dysplasia to advanced lung adenocarcinomas induced by c-Raf-1. PLoS ONE.

[7] Guinney J., Dienstmann R., Wang X., de Reyniès A., Schlicker A., Soneson C., Marisa L., Roepman P., Nyamundanda G., Angelino P. (2015). The consensus molecular subtypes of colorectal cancer. Nat. Med..

[8] Dreyer C., Afchain P., Trouilloud I., André T. (2016). New molecular classification of colorectal cancer, pancreatic cancer and stomach cancer: Towards “à la carte” treatment?. Bull. Cancer.

[9] Komor M.A., Bosch L.J., Bounova G., Bolijn A.S., Delis-van Diemen P.M., Rausch C., Hoogstrate Y., Stubbs A.P., de Jong M., Jenster G. (2018). Consensus molecular subtype classification of colorectal adenomas. J. Pathol..

[10] Liu W., Kuang T., Liu L., Deng W. (2024). The role of innate immune cells in the colorectal cancer tumor microenvironment and advances in anti-tumor therapy research. Front. Immunol..

[11] Xing C., Du Y., Duan T., Nim K., Chu J., Wang H.Y., Wang R.F. (2022). Interaction between microbiota and immunity and its implication in colorectal cancer. Front. Immunol..

[12] Han B., Takvorian P.M., Weiss L.M. (2020). Invasion of host cells by microsporidia. Front. Microbiol..

[13] Asghari A., Mohammadi M.R., Norouzi R., Heidari S., Kakian F., Pouryousef A., Badri M., Maleki F., Pour M.K. (2026). A global overview of microsporidia infection in HIV/AIDS patients: An updated systematic review and meta-analysis. Int. Health.

[14] Redondo F., Hurtado-Marcos C., Izquierdo F., Cuéllar C., Fenoy S., Sáez Y., Magnet Á., Galindo-Regal L., Uribe N., López-Bañeres M. (2022). Latent microsporidia infection prevalence as a risk factor in colorectal cancer patients. Cancers.

[15] Andreu-Ballester J.C., Galindo-Regal L., Hidalgo-Coloma J., Cuéllar C., García-Ballesteros C., Hurtado C., Uribe N., Martín M.C., Jiménez A.I., López-Chuliá F. (2020). Differences in circulating γδ T cells in patients with primary colorectal cancer and relation with prognostic factors. PLoS ONE.

[16] Pan L., Zhou Y., Kuang Y., Wang C., Wang W., Hu X., Chen X. (2024). Progress of research on γδ T cells in colorectal cancer (Review). Oncol. Rep..

[17] Andreu-Ballester J.C., Amigó-García V., Catalán-Serra I., Gil-Borrás R., Ballester F., Almela-Quilis A., Millan-Scheiding M., Peñarroja-Otero C. (2011). Deficit of gammadelta T lymphocytes in the peripheral blood of patients with Crohn’s disease. Dig. Dis. Sci..

[18] Bernstein C.N., Blanchard J.F., Kliewer E., Wajda A. (2001). Cancer risk in patients with inflammatory bowel disease: A population-based study. Cancer.

[19] Pedersen N., Duricova D., Elkjaer M., Gamborg M., Munkholm P., Jess T. (2010). Risk of extra-intestinal cancer in inflammatory bowel disease: Meta-analysis of population-based cohort studies. Am. J. Gastroenterol..

[20] Andreu-Ballester J.C., Garcia-Ballesteros C., Amigo V., Ballester F., Gil-Borrás R., Catalán-Serra I., Magnet A., Fenoy S., del Aguila C., Ferrando-Marco J. (2013). Microsporidia and its relation to Crohn’s disease: A retrospective study. PLoS ONE.

[21] Andreu-Ballester J.C., Hurtado-Marcos C., García-Ballesteros C., Pérez-Griera J., Izquierdo F., Ollero D., Jiménez A., Gil-Borrás R., Llombart-Cussac A., López-Chuliá F. (2025). Decreased gene expression of interleukin 2 receptor subunit γ (CD132) in tissues of patients with Crohn’s disease. World J. Gastroenterol..

[22] Izquierdo F., Moura H., Bornay-Llinares F.J., Sriram R., Hurtado C., Magnet Á., Fenoy S., Visvesvara G., Del Aguila C. (2017). Production and characterization of monoclonal antibodies against *Encephalitozoon intestinalis* and *Encephalitozoon* sp. spores and their developmental stages. Parasites Vectors.

[23] Accoceberry I., Thellier M., Desportes-Livage I., Achbarou A., Biligui S., Danis M., Datry A. (1999). Production of monoclonal antibodies directed against the microsporidium *Enterocytozoon bieneusi*. J. Clin. Microbiol..

[24] Polley S.D., Boadi S., Watson J., Curry A., Chiodini P.L. (2011). Detection and species identification of microsporidial infections using SYBR Green real-time PCR. J. Med. Microbiol..

[25] Moerk K., Lokau J. (2026). Molecular regulation of common γ-chain/interleukin-2 family cytokine receptors. Biochim. Biophys. Acta Mol. Cell Res..

[26] Zuo D.B., Chen Z.H., Jin Y.M., Sang M., Sun X.D., Guo A., Li X.Y., Wu J.X., Ji K.K., Zhou H. (2026). Common gamma chain cytokines-driven optimization of chimeric antigen receptor T cells: Mechanistic insights and future directions. World J. Clin. Oncol..

[27] Jacovas V.C., Zelnick M., McNulty S., Ross J.E., Khurana N., Pan X., Nieto A., Martin S., McLean B., Elnagheeb M.A. (2026). The ClinGen severe combined immunodeficiency disease variant curation expert panel: Specifications for classification of variants in ADA, DCLRE1C, IL2RG, IL7R, JAK3, RAG1, and RAG2. Genet. Med..

[28] Briassouli E., Marinakis N., Spoulou V., Notarangelo L.D. (2026). IL2RG-related immunodeficiencies: From SCID to atypical presentations. Front. Immunol..

[29] Cui C., Zhang T.-T., Lin Q., Huang T.X., Rao E.Y., Du J.H., Fu L. (2024). WNT2 blockade augments antitumor immunity by attenuating myeloid-derived suppressor cells in colorectal cancer. MedComm–Oncology.

[30] Leonard C.A., Schell M., Schoborg R.V., Hayman J.R. (2013). *Encephalitozoon intestinalis* infection increases host cell mutation frequency. Infect. Agents Cancer.

[31] del Aguila C., Izquierdo F., Granja A.G., Hurtado C., Fenoy S., Fresno M., Revilla Y. (2006). *Encephalitozoon* microsporidia modulates p53-mediated apoptosis in infected cells. Int. J. Parasitol..

